# Fabrication and Characteristics of SnAgCu Alloy Nanowires for Electrical Connection Application

**DOI:** 10.3390/mi9120644

**Published:** 2018-12-05

**Authors:** Jung-Hsuan Chen, Shen-Chuan Lo, Shu-Chi Hsu, Chun-Yao Hsu

**Affiliations:** 1 Department of Industrial Education, National Taiwan Normal University, 106 Taipei, Taiwan; 2 Department of Electron Microscopy Development Application, Industrial Technology Research Institute, 310 Hsinchu, Taiwan; SCLo@itri.org.tw (S.-C.L.); itri4907@itri.org.tw (S.-C.H.); 3 Department of Mechanical Engineering, Lunghwa University of Science and Technology, 333 Taoyuan, Taiwan

**Keywords:** SAC305, solder, nanowires, intermetallic compound, electrical connection

## Abstract

As electronic products become more functional, the devices are required to provide better performances and meet ever smaller form factor requirements. To achieve a higher I/O density within the smallest form factor package, applying nanotechniques to electronic packaging can be regarded as a possible approach in microelectronic technology. Sn-3.0 wt% Ag-0.5 wt% Cu (SAC305) is a common solder material of electrical connections in microelectronic devices. In this study, SAC305 alloy nanowire was fabricated in a porous alumina membrane with a pore diameter of 50 nm by the pressure casting method. The crystal structure and composition analyses of SAC305 nanowires show that the main structure of the nanowire is β-Sn, and the intermetallic compound, Ag_3_Sn, locates randomly but always appears on the top of the nanowire. Furthermore, differential scanning calorimetry (DSC) results indicate the melting point of SAC305 alloy nanowire is around 227.7 °C. The melting point of SAC305 alloy nanowire is significantly higher than that of SAC305 bulk alloy (219.4 °C). It is supposed that the non-uniform phase distribution and composite difference between the nanowires causes the change of melting temperature.

## 1. Introduction

Electronic packaging is the final manufacturing process to transform semiconductor devices into functional products and advanced packaging has a direct impact on product performance and success rates [1,2]. As electronic and mobile devices continue to develop, the devices are required to meet the demand of ultra-thin, ultra-light, high performance, and low power consumption. Therefore, advanced 3D microelectronic packaging technology is becoming the industry trend [3,4,5,6]. More and more Input/Output (I/O) pins are needed in electronic packaging of integrated circuits while pitch is getting smaller and smaller. In order to achieve fine pitch requirement, applying nanotechniques to electronic packaging can be regarded as a possible approach in microelectronic technology. In recent years, nano-sized interconnections for advanced packaging have drawn much attention from researchers and scientists. Nanosolder is necessary to performed the soldering or bonding at the nanoscale.

Among the numerous solder materials, tin-silver-copper (SAC) based alloys are widely used to replace the conventional tin-lead solders and have advantages such as low melting temperature, superior mechanical properties, and good solderability [7,8]. Nanoscale SAC solder alloys [9,10,11,12,13,14,15,16] such as nanoparticles and nanowires had been prepared in these years. SAC alloy nanoparticles could be produced by chemical methods and physical methods, such as electrodeposition, chemical reduction technique, consumable-electrode direct current arc technique, etc. These nanoparticles were easy to aggregate yet difficult to handle and perform soldering directly. SAC alloy nanowires such as Sn_88_Ag_5_Cu_7_, Sn_93_Ag_4_Cu_3_, Sn_58_Ag_18_Cu_24_, Sn_78_Ag_16_Cu_6_, Sn_90_Ag_4_Cu_6_, Sn_87_Ag_4_Cu_9_ alloy nanowires were fabricated by electrodeposition using various values of the deposition potential [16]. However, SAC305 alloy, a common commercial lead-free solder alloy, has not been produced in the form of nanowire until now. 

In this study, we fabricated SAC305 alloy nanowires inside the porous alumina membrane by the pressure casting method. The structural and thermal characteristics of SAC305 alloy nanowires were investigated and the differences from SAC305 bulk alloy were studied. Furthermore, the nanowire array in the soldering application was also discussed in this work.

## 2. Materials and Methods

**Figure 1:**
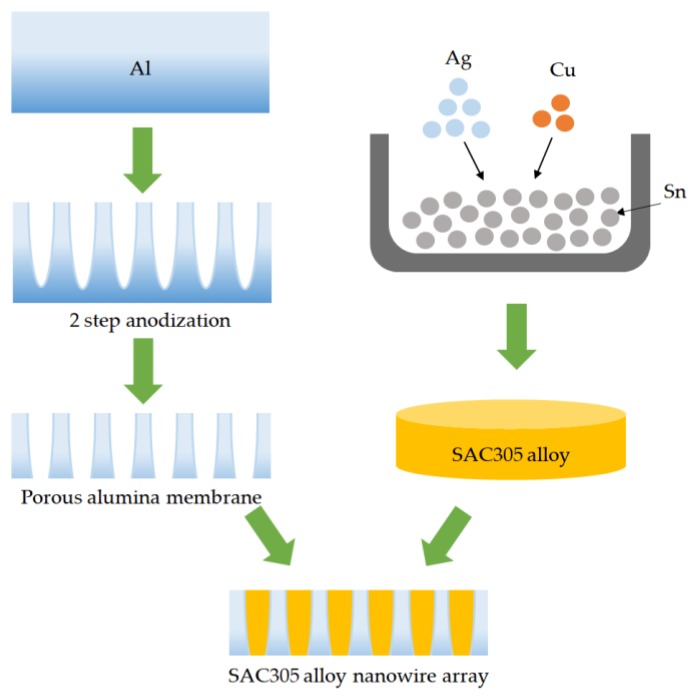
Schematic process flow of SAC305 alloy nanowires fabrication in this study.

Solder alloy nanowire array was fabricated in a porous alumina membrane with a pore diameter around 50 nm by the pressure casting method. The processes were divided into three parts: production of the porous alumina template, smelting of the solder alloy, and fabrication of the alloy nanowires. The detail process flow is shown in Figure 1. Firstly, the porous alumina template was fabricated using a two-step anodization. An Al sheet (purity 99.7%) was anodized in 0.3 M oxalic acid solution (H_2_C_2_O_4_) at 20 °C for 1 h. Then, the alumina film formed by the first anodization was removed in a solution, which was composed of the phosphoric acid (H_3_PO_4_) and the chromic acid (H_2_CrO_4_) at 60 ^o^C. The second anodization was performed using the condition of the first anodization. After removing the Al substrate and the barrier layer by 0.1 M sodium hydroxide solution (NaOH), the porous alumina membrane was produced.

Secondly, pure tin (Sn) particles (purity 99.99%), pure silver (Ag) particles (purity 99.99%), and pure cupper (Cu) particles (purity 99.99%) were well mixed in a quartz crucible. The weights of the three pure metals (Sn, Ag, and Cu) were in the ratio of 96.5:3.0:0.5. After that, the quartz crucible was heated to 1100 °C in vacuum. The alloy, Sn-3.0 wt% Ag-0.5 wt% Cu (SAC305), was formed after cooling to room temperature.

Finally, SAC305 alloy nanowire array was produced using the high vacuum pressure casting technique. The experimental equipment and principle were described in detail in our previous studies [17,18]. The porous alumina membrane produced in this work had a thickness of 10 μm and the area of the membrane was 1cm^2^. A disc shaped piece of SAC305 alloy with dimensions of 1 cm^2^ × 0.1 cm was placed on the top of the porous alumina membrane inside the chamber. The vacuum pressure of the chamber was maintained at 10^−6^ Torr to prevent the metal oxidation. After the chamber was heating to 300 °C for 20 min, a hydraulic force was applied to SAC305 alloy melt. During casting process, the molten alloy was injected into the nanochannel of the porous alumina membrane. Solidification was performed using water quenching at the bottom of the chamber. After cooling to room temperature, SAC305 alloy nanowire array was produced.

The crystal structure, phase distribution, and composition of SAC305 alloy nanowires were examined by X-ray diffraction (XRD, Bruker, Billerica, MA, USA) scanning electron microscope (SEM, JEOL, Tokyo, Japan), energy dispersive spectroscopy (EDS, Oxford, Abingdon, UK), and transmission electron microscope (TEM, JEOL, Tokyo, Japan). Moreover, the melting point and thermal properties of SAC305 alloy nanowires were measured by differential scanning calorimetry (DSC, TA, New Castle, DE, USA) analysis.

## 3. Results and Discussion

### 3.1. Fabrication of SAC305 Alloy Nanowires

**Figure 2:**
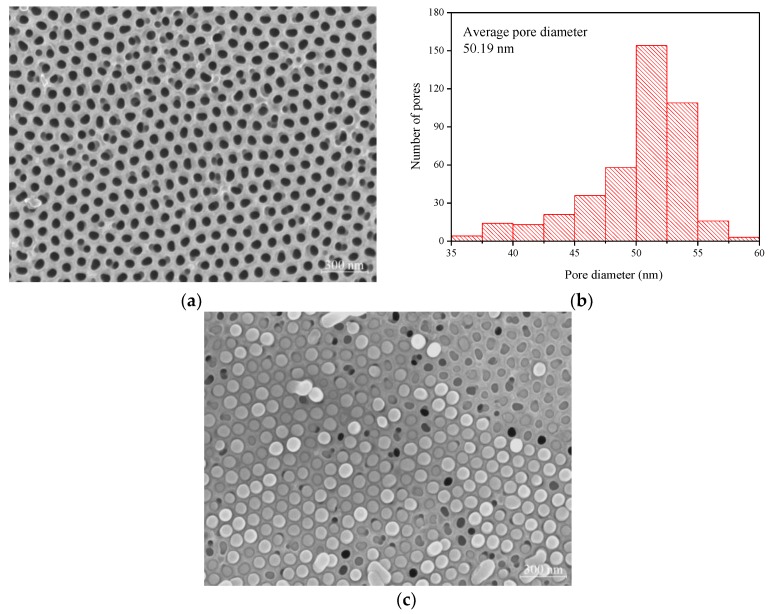
(**a**) SEM image of the porous alumina membrane; (**b**) The pore size distribution which was calculated from image (**a**) and the average pore diameter denoted in the figure; (**c**) SEM image of SAC305 alloy nanowires in the porous alumina membrane.

In this study, SAC305 nanowires were fabricated in the porous alumina membrane by the pressure casting process. Figure 2a shows that the surface morphology of the porous alumina membrane produced in the oxalic acid solution reveals that the nanopores are order and uniform arrays. The SEM image of the porous alumina membrane is further analyzed with the image pro plus software. The corresponding pore size distribution was obtained by the histogram analysis. As presented in Figure 2b, the histogram was plotted by measuring at least 400 pores in Figure 2a and the average pore diameter is about 50.19 nm. Figure 2c shows the plane view of SAC305 alloy nanowires in the porous alumina membrane. A high filling ratio of nanowire could be achieved by the pressure casting process.

### 3.2. Characteristics of SAC305 Alloy Nanowires

**Figure 3:**
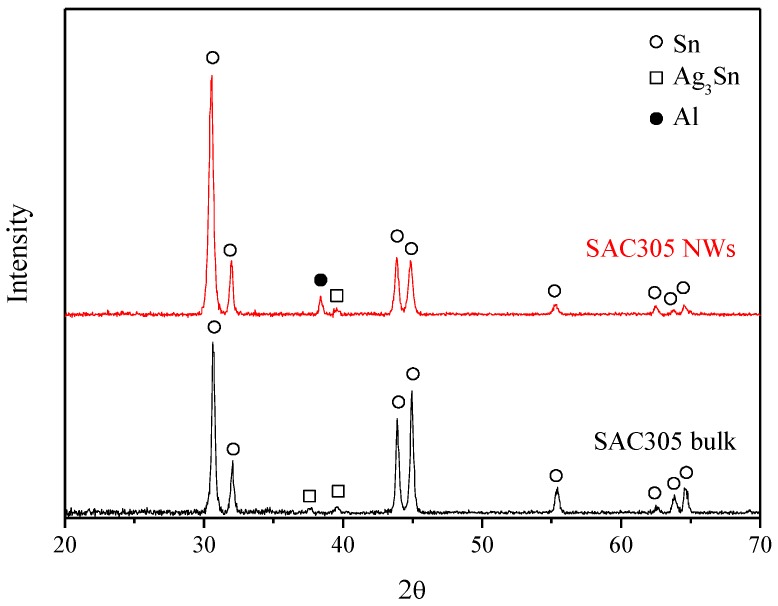
XRD spectra of SAC305 alloy and SAC305 alloy nanowires.

X-ray diffraction analyses of SAC305 bulk alloy and nanowires are shown in Figure 3. The signals of Sn in SAC305 bulk alloy and nanowires are identified as beta-Sn (β-Sn). The β-Sn phase crystallizes in a tetragonal crystal structure (I4_1_/and, space group = 141, JCPDS No. 04-0673) with the lattice constants, a = 0.5831 nm and c = 0.3182 nm. The major crystal plane of β-Sn in SAC305 bulk alloy and nanowires is the (200) lattice plane locating at the angle of 30.6°. Some slight signals form Ag_3_Sn are also observed. The results indicate that the crystal structure of SAC305 bulk alloy is preserved in the nanowires after heating and high-pressure processes. Additionally, the diffraction peak of Al in SAC305 alloy nanowires is coming from the residual Al substrate of the porous alumina membrane.

**Figure 4:**
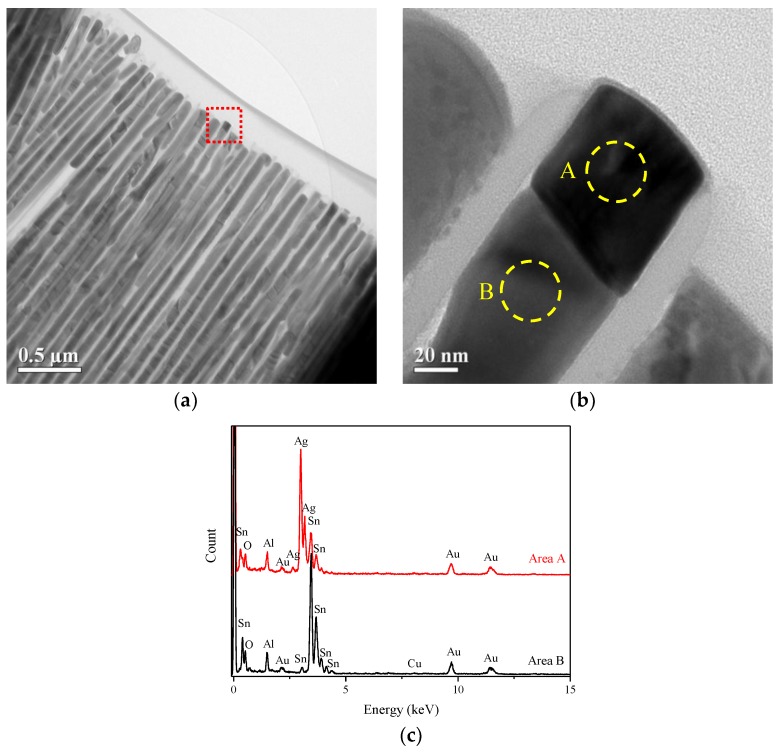
(**a**) Low magnification TEM image of SAC305 alloy nanowires; (**b**) The enlarged image of the red square in the image (**a**); (**c**) Energy dispersive X-ray spectra recorded from the circular areas in (**b**).

**Figure 5:**
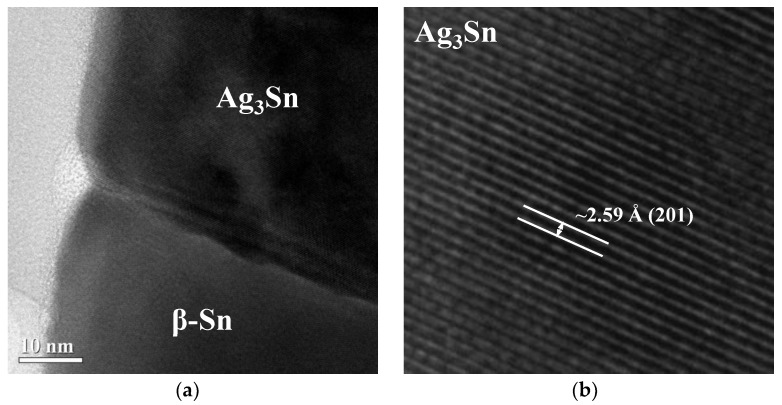

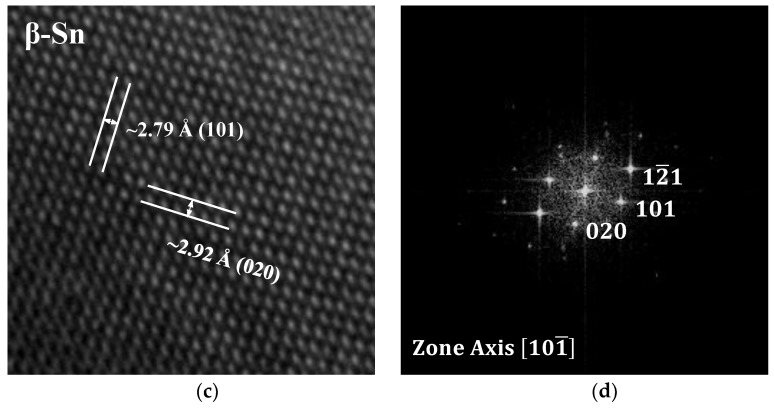
(**a**) TEM image of the interface between Ag_3_Sn and β-Sn. (**b**) High-resolution TEM image of Ag_3_Sn crystal. (**c**) High-resolution TEM image of β-Sn crystal. (**d**) FFT analysis of the lattice fringes in (**c**).

The low-magnification cross-sectional structure of SAC305 alloy nanowires is presented in the bright field TEM image as shown in Figure 4a. Figure 4b, which is the enlarged image of the red box in Figure 4a, reveals a significant contrast difference and interface in the upper region of the nanowire. EDS analysis was performed with field emission TEM to determine the composition of nanowire marked in Figure 4b. From the EDS spectrum, the crystal on the top of the nanowire (area a) is judged to consist of Ag and Sn in the ratio of 3:1 with a statistical error of 5%. The gold signal is coming from the TEM gold grid. Area b is mainly composed of Sn, but a low Cu content around 0.5 wt% is also observed. High-resolution TEM analyses were performed to determine the crystal structure of the nanowire. Figure 5b is taken from the top of the nanowire. It indicates that the lattice fringes (d = 2.59 Å) observed in the high-resolution TEM image is identical with the distance between the (201) lattice plane and confirms that the nanocrystal is Ag_3_Sn. The high-resolution TEM (Figure 5c) of the region under the Ag_3_Sn crystal in the nanowire shows that the lattice fringes, d = 2.92 Å and d = 2.79 Å, are identical to the distance between the (200) lattice plane and (101) lattice plane of β-Sn, respectively. Fast Fourier-Transformation (FFT) analysis was performed on the lattice fringes of Figure 5c and the spots with a zone axis [10$\bar{1}$] match well with the tetragonal structure of β-Sn as shown in Figure 5d. According to the TEM and EDS results, the nanowire is mainly composed of β-Sn. Furthermore, a Ag_3_Sn crystal always appears on the top of the nanowire and has a non-uniform distribution in the nanowire arrays. However, the intermetallic compounds composed of Sn and Cu such as Cu_6_Sn_5_ and Cu_3_Sn were not found in our experiment. It is supposed that the reason for this is that Cu_6_Sn_5_ or Cu_3_Sn has a smaller crystal size and much lower content than Ag_3_Sn, causing difficulty in observation.

**Figure 6:**
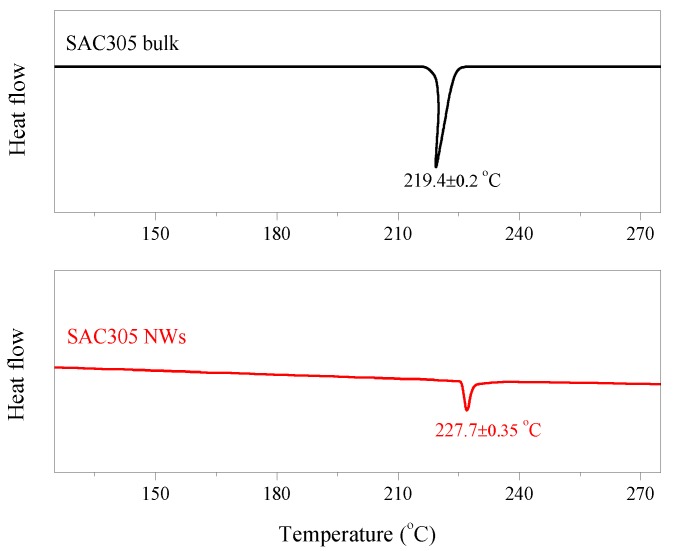
Differential scanning calorimetry (DSC) thermographs of SAC305 alloy and SAC305 alloy nanowires.

The DSC curves in Figure 6 show that there is a variation in the melting point between SAC305 bulk alloy and the nanowires. The melting temperature increases from 219.4 °C to 227.7 °C as the alloy’s crystal size decreases. For nanocrystals embedded in a matrix, they can melt below the melting point of the bulk materials when the interfaces between embedded nanocrystals and the matrix are incoherent [19]. Moreover, the melting point depends strongly on the surface to volume ratio in the nanoscale range [20]. Our experimental result does not agree with the theoretical prediction but is just the reverse. This could be explained by the fact that not only the crystal size decreases but also the composition and phase distribution change during the formation of the nanowire. TEM and EDS results indicate that the intermetallic compound, Ag_3_Sn, always appears on the top of the nanowires and the composition of the nanowire except Ag_3_Sn crystal is mainly composed of Sn and Cu (~0.5 wt%). According to the Sn–Ag–Cu ternary phase diagram [21], the melting point of SAC 305 alloy is around 220 °C and the melting point of SAC alloy increases as the decrease of Ag content. Furthermore, Ag diffusion during the heating process might become difficult because of the confinement of the nanochannel in the porous alumina membrane. Therefore, the melting behavior of SAC305 alloy nanowire is more similar to the Sn–Cu system rather than the Sn–Ag–Cu system, leading to the higher melting point than SAC305 bulk alloy.

**Figure 7:**
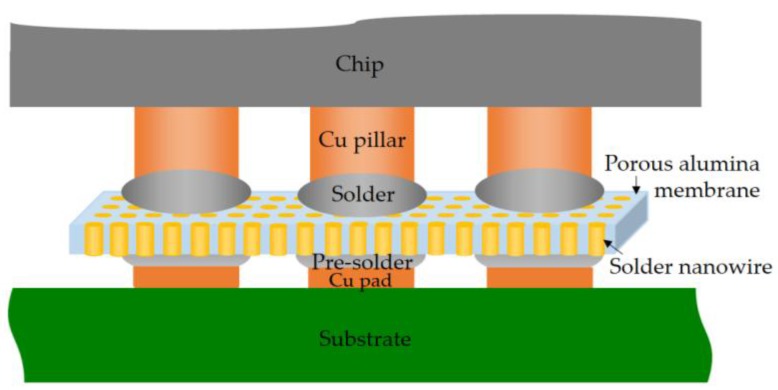
Schematic illustration of an electrical connection structure with the solder alloy nanowires.

Fine pitch and submicron bump technology is a possible way to achieve a higher I/O density within the smallest form factor package. In this study, we propose a new joint structure with the solder alloy nanowire array as shown in Figure 7 for the submicron electronic packaging. The solder alloy nanowires are the bridges between the solder cap on the Cu pillar and the pre-solder of the substrate or another chip. Some advantages such as offering more overlay budget and reducing the common soldering problems like cold joints and solder bridges could be obtained using the new joint structure. Although there are still concerns about reliability and mechanical stress issues, this technique could be helpful to develop the interconnection technology for the advanced packaging where high-density joint interconnection is required. Additionally, more stable and more flexible nanostructured templates with the suitable coefficient of thermal expansion (CTE) would be a good candidate reducing the joint stress for the practical application [22,23].

## 4. Conclusions

SAC305 alloy nanowires have been successfully fabricated inside the porous alumina membrane by the pressure casting method. The nanowires with high aspect ratio are arranged uniformly. Experimental results show that the intermetallic compound, Ag_3_Sn, locates randomly but always appears on the top of the nanowire. The composition of the nanowire, except Ag_3_Sn crystal, is mainly composed of Sn and Cu (~0.5 wt%). DSC results indicate that SAC305 alloy nanowire has a higher melting temperature than SAC305 bulk alloy. The melting point increase from 219.4 °C to 227.7 °C might be caused by the non-uniform phase distribution and composition difference. Finally, we propose a possible joint structure in which the solder alloy nanowires could provide an electrical connection between chip and substrate or chip and chip to achieve the high-density joint interconnection.
